# Foraging behavior and locomotion of the invasive Argentine ant from winter aggregations

**DOI:** 10.1371/journal.pone.0202117

**Published:** 2018-08-09

**Authors:** Benjamin P. Burford, Gail Lee, Daniel A. Friedman, Esmé Brachmann, Rebia Khan, Dylan J. MacArthur-Waltz, Aidan D. McCarty, Deborah M. Gordon

**Affiliations:** 1 Department of Biology, Hopkins Marine Station, Stanford University, Pacific Grove, California, United States of America; 2 Department of Biology, Stanford University, Stanford, California, United States of America; 3 College of Letters & Science, University of California Berkeley, Berkeley, California, United States of America; University of Sheffield, UNITED KINGDOM

## Abstract

The collective behavior of ant colonies, and locomotion of individuals within a colony, both respond to changing conditions. The invasive Argentine ant (*Linepithema humile*) thrives in Mediterranean climates with hot, dry summers and colder, wet winters. However, its foraging behavior and locomotion has rarely been studied in the winter. We examined how the foraging behavior of three distinct *L*. *humile* colonies was related to environmental conditions and the locomotion of workers during winter in northern California. We found that colonies foraged most between 10 and 15°C, regardless of the maximum daily temperature. Worker walking speed was positively associated with temperature (range 6–24°C) and negatively associated with humidity (range 25–93%RH). All colonies foraged during all day and night hours in a predictable daily cycle, with a correlation between the rate of incoming and outgoing foragers. Foraging activity was unrelated to the activity of a competing native ant species, *Prenolepis imparis*, which was present in low abundance, and ceased only during heavy rain when ants left foraging trails and aggregated in small sheltered areas on trees.

## Introduction

The invasive Argentine ant, *Linepithema humile*, is established in Mediterranean climates worldwide, where it is unicolonial and seasonally polydomous. Within a colony, workers, queens, and brood are distributed among multiple nests linked by a trail network that expands and contracts seasonally. During the polydomous summer phase, many workers can quickly recruit to nutritional resources directly from the colony’s vast trail network [1], and then use the network to distribute resources among multiple nests [2]. Colonies condense during winter in Mediterranean climates, to aggregate in a single nest with very few foraging trails [3–5]. Winter conditions, including precipitation, cool temperatures, and decreased sunlight, reduce resource availability relative to summer conditions [6, 7]. However, most studies of the influence of environmental conditions on foraging behavior and locomotion in the invaded range have been made in warm, dry summer conditions. Here we consider the behavior of *L*. *humile* in winter conditions in northern California to further examine how it competes with native species in invaded habitats.

*Linepithema humile* forage collectively, a process that in many ant species is regulated by interactions among individuals [8]. As poikilotherms, the speed at which ants are able to move and interact increases with ambient temperature [9–11]. Foraging behavior is also adjusted in association with temperature [12–18], and this relation depends on biotic factors such as food availability, habitat structure, and interactions with other species [15, 16, 19–21]. *Linepithema humile* are active under a wide range of conditions, but the highest sustained rates of foraging recorded in the invasive range occur during summer, when temperatures range between 10 and 35°C [22–26]. Foraging by *L*. *humile* during this season declines outside the optimal temperature range [22–26], which may allow some native ant species to persist in invaded habitats [26–28]. In addition, locomotion rate in *L*. *humile* declines with temperature [11], so that cooler conditions reduce the thermal envelope for foraging [29]. Although starvation and cool temperatures do not reduce resource discovery by *L*. *humile* colonies examined in laboratory settings [30], the narrow mid-winter temperature requirement (7–14°C) that appears necessary for this species to become established in a given habitat [31–33] suggests that foraging is restricted by temperature.

**Fig 1:**
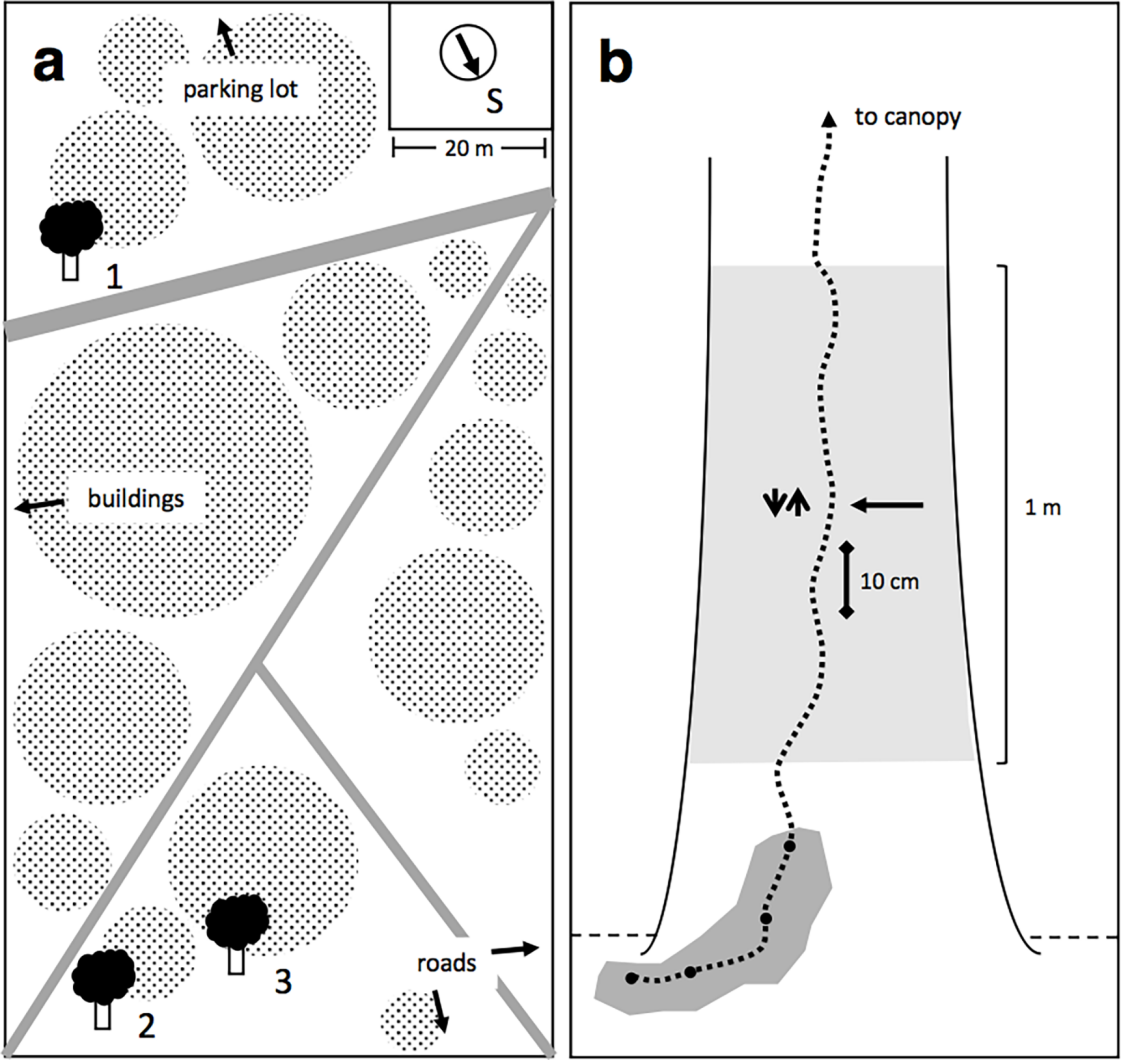
The study area and observational methods at each *Linepithema humile* colony. In the study area (a) on Stanford main campus, numbers represent locations of nests, grey lines paved paths, and shaded circles canopy cover. We consider colonies 2 and 3 to be distinct colonies in spite of their spatial proximity because we never saw trails connecting the two trees. In the observational methods at each tree (b), points represent entrances to the nest (dark shaded area); dashed lines the bidirectional foraging trail; the light shaded area the trunk area where *Prenolepis imparis* were counted; dark horizontal arrow the line where *L*. *humile* foraging rate was recorded; and the 10.0 cm line parallel to the trail the line where *L*. *humile* walking speed was measured.

Winter daily patterns of foraging and locomotion are known from only a single study in southern California [22, 34]. In northern California, the native winter ant, *Prenolepis imparis*, is one of the few native ant species remaining in urban environments where Argentine ants are present [35]. It is active mainly in the winter [36], competes with *L*. *humile* for food resources [37, 38], and has a chemical defense effective against *L*. *humile* [39]. To examine the behavior of *L*. *humile* under wintertime conditions, and in the presence of *P*. *imparis*, we recorded the rate and speed at which ants travelled on foraging trails of three winter aggregations in northern California (Fig 1A) for three 24-hour periods, and concurrently measured ambient temperature, relative humidity, light level, and the abundance of *P*. *imparis*. Our results 1) show evidence for a winter circadian rhythm, 2) describe a shelter-seeking behavior in response to heavy rain, 3) indicate that low numbers of a competing ant species do not deter foraging by *L*. *humile*, and 4) demonstrate that locomotion and foraging activity differ in relation to temperature and humidity.

## Results

### Colony foraging behavior

**Fig 2:**
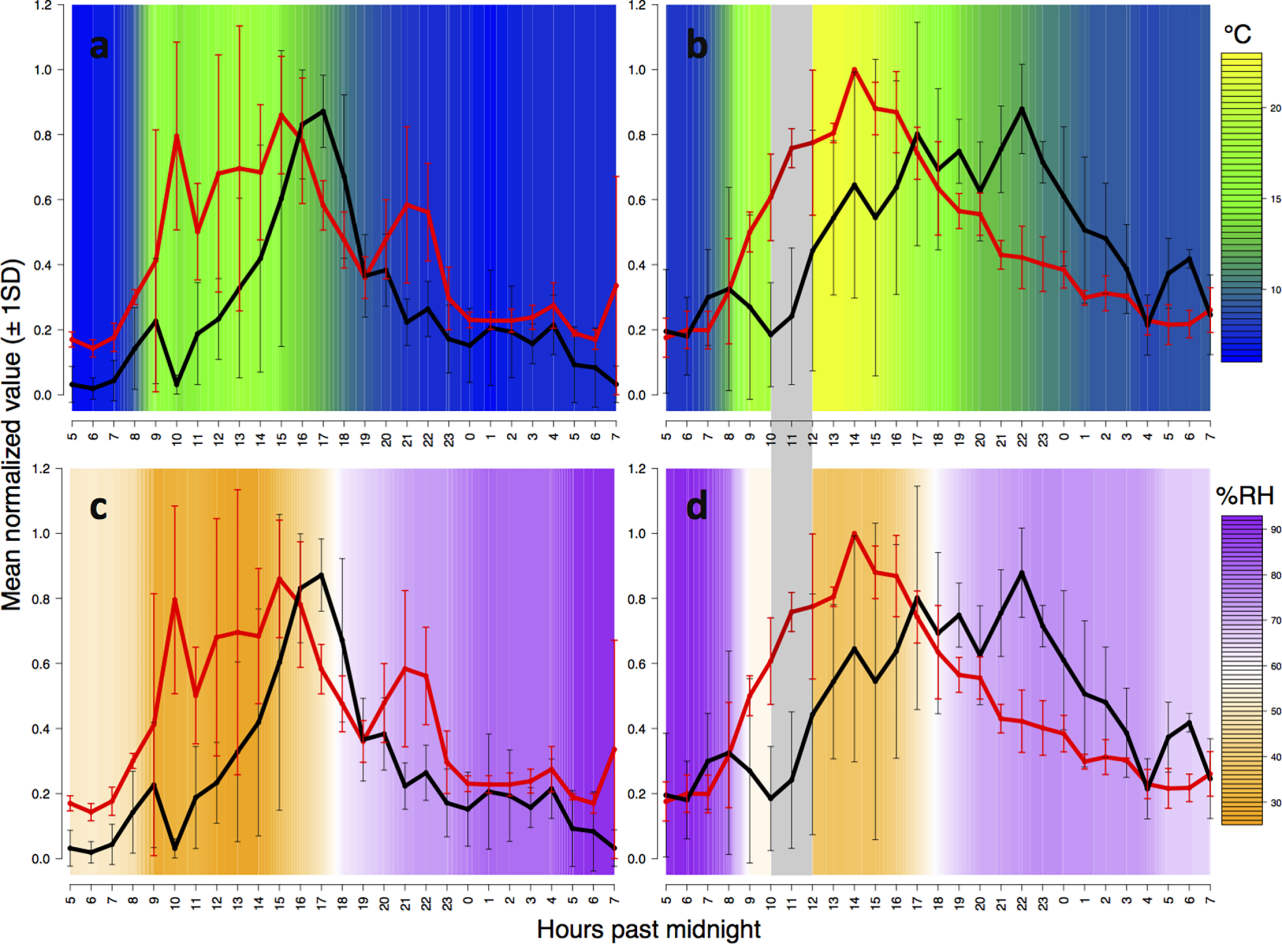
Walking speed and foraging rate in relation to temperature and humidity. Average normalized (0–1 scale) hourly foraging rate (black lines) and walking speed (red lines) ± 1SD are plotted against linearly-interpolated average hourly temperature (°C; a and b) and humidity (%RH; c and d) on the coolest (a and c) and warmest (b and d) observations. Temperature and humidity data are missing from 10:00–12:00 on the warmest observation (shaded in grey) due to a temporary failure of power supply in the sensor.

**Fig 3:**
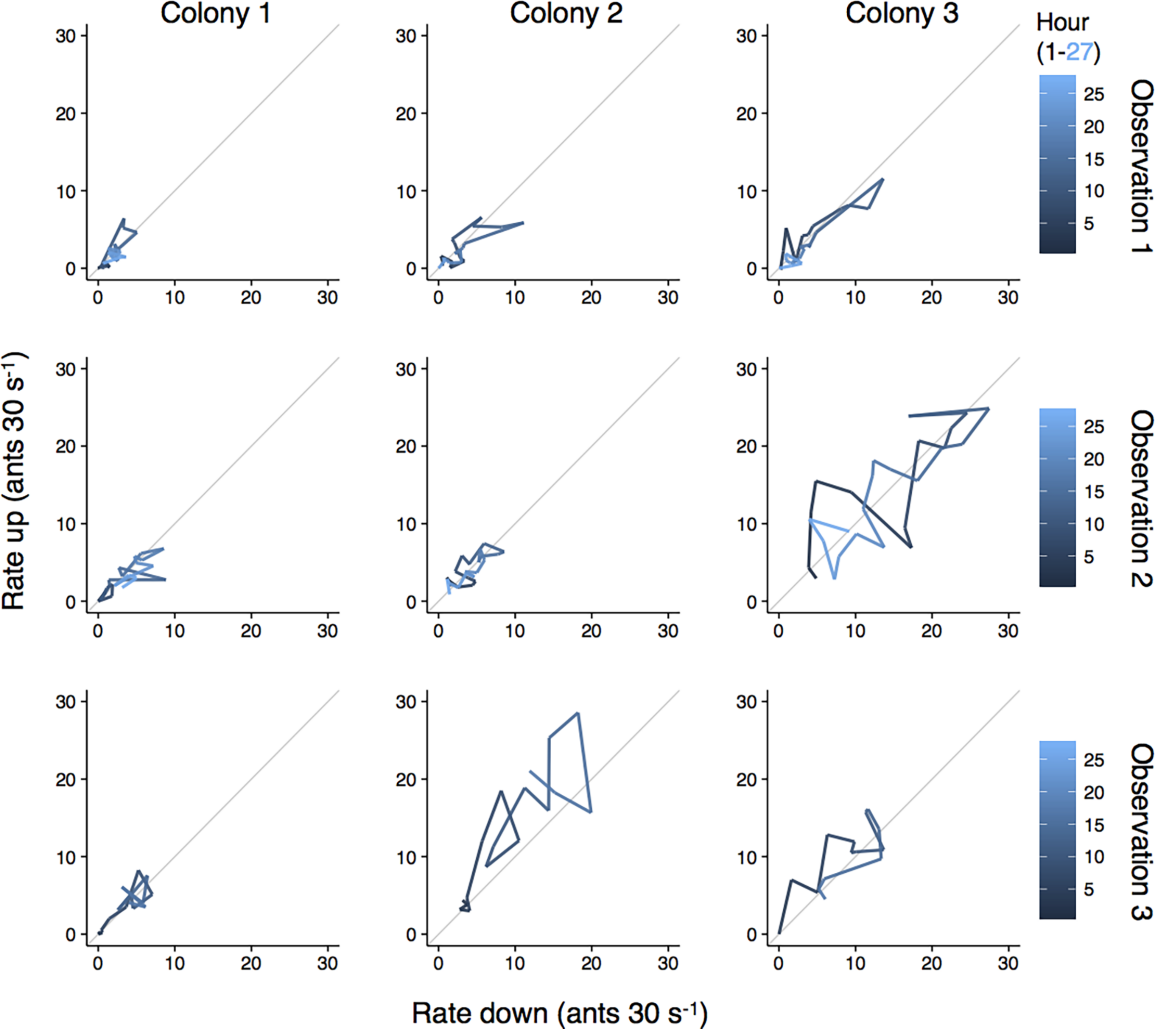
The temporal pattern of *Linepithema humile* colony foraging activity in the rate of returning and outgoing foragers. Line color represents time since observation start: during observations 1 and 2, hour 1 and 27 are equal to 05:00 on day 1 and 07:00 on day 2 (PST), respectively. The third observation occurred from hour 2 to 18, or 06:00 to 23:00 on the same day. Grey lines represent a slope of 1.

**Fig 4:**
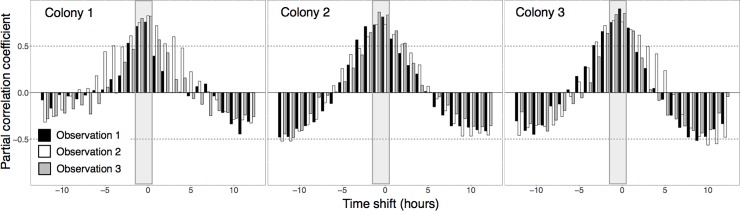
The correlation between returning and outgoing *Linepithema humile* foragers. Bars represent partial correlation coefficients between normalized (0–1 scale) hourly foraging rate down and up trees when the foraging rate up is shifted -12 to 12 hours. Partial correlation coefficients were greatest with a lag of 0–1 hours between rates down and rates up (shaded in grey).

All three *L*. *humile* winter aggregations maintained consistent bidirectional foraging trails on *Quercus agrifolia* (Coast live oak) trees, with maximum sustained foraging rates ranging from 0.29–0.95 ants s^-1^ and forager walking speeds from 1.85–2.46 cm s^-1^. In all observations, foragers traveling up trees rarely had full abdomens, while most traveling down did; this suggests that ants were harvesting honeydew from scale insects observed in canopy foliage [7, 22, 40, 41]. The rate of ants travelling in both directions on trails was low in the early morning, rapidly increased until early or late afternoon, and then gradually returned to the low early morning level (Fig 2). During all hours of the 24-hour cycle of foraging, each colony maintained an approximately equal rate of ants leaving and returning to the nest (Fig 3). The foraging rate up trees was positively correlated with the foraging rate down trees at a lag of 1 hour, and to a lesser extent, up to a lag of 3 hours (partial correlation coefficients from autoregressive models > 0.5; Fig 4). Thus, the rate of outgoing foragers exhibited the highest association with the rate of foragers returning to the nest the hour of or the hour before. Foraging trails dissolved only during heavy rain, when foragers aggregated in sheltered areas on tree trunks near the trail instead of returning to the nest. These small aggregations were isolated from each other by water on the tree. When the rain stopped, foragers moved between sheltered areas and, once the trunk dried further, the foraging trail was reformed.

### Environmental conditions, walking speed, and foraging

**Fig 5:**
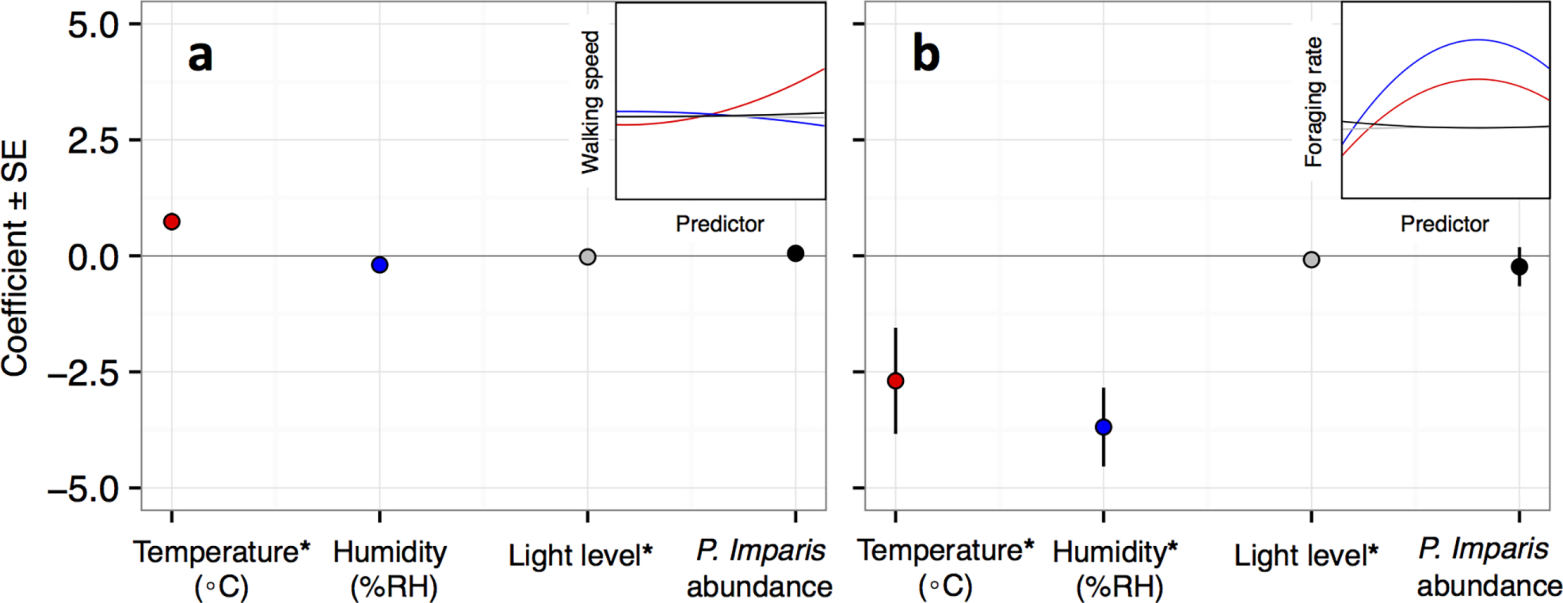
Analysis of the association of environmental factors with log transformed *Linepithema humile* (a) walking speed and (b) foraging rate. Points represent coefficients (± SE) of the x^2^ term from quadratic mixed effects models, and are color-coded by predictor: red = temperature, blue = humidity, grey = light level, and black = *P*. *imparis* abundance. Error bars in (a) are present, but small. A Bonferroni correction reduces alpha to 0.013, and asterisks (*) on the x-axis labels represent significant associations. Corrections for covariance between temperature and humidity resulted in the following coefficients (all p < 0.013): walking speed-temperature (0.62), walking speed-humidity (-0.38), foraging rate-temperature (-2.60), and foraging rate-humidity (-3.36). Insets in both (a) and (b) are visualizations of the quadratic equations (based on slopes and intercepts) relating each dependent and independent variable from the mixed effects models.

Ambient temperature, which ranged between 6.3 and 24°C, was strongly associated with tree surface temperature (linear regression, p < 0.003). Ant walking speed was positively related to ambient temperature (quadratic mixed effect model, p < 0.003; Fig 5A); an increase of 1°C corresponded to an average (± 1SD) increase of 0.1 (± 0.03) cm s^-1^ in walking speed. Colony foraging rate showed a negative parabolic relationship with ambient temperature (quadratic mixed effect model, p = 0.003; Fig 5B). Foraging activity was highest for 5–6 hours at temperatures from 10–15°C in the evening (Fig 2). These thermal conditions also occurred for about 1 hour during the morning without a surge in colony foraging effort. Walking speed was not significantly related to humidity, but showed a mildly negative association (Fig 5A); an increase of 1% RH corresponded to an average (± 1SD) decrease of 0.013 (± 0.012) cm s^-1^ in walking speed. Accounting for covariation between temperature and humidity showed that walking speed had a significant association with humidity and a smaller coefficient, while the association with temperature remained significant and also had a smaller coefficient (Fig 5). Colony foraging rate showed a negative parabolic association with humidity (quadratic mixed effects model, p < 0.003; Fig 5B); when accounting for covariation of predictors, both temperature and humidity remained significant in their association with foraging rate, and coefficients became marginally smaller (Fig 5). Light level showed a significant neutral association with walking speed and foraging rate (quadratic mixed effects models, p < 0.003 in both cases; Fig 5A and 5B). Neither walking speed nor foraging rate were associated with the abundance of *P*. *imparis* (ranged from 0–5), which was seen often at Colony 2, less at Colony 1, and never at Colony 3. When temperatures were lowest (observation 1), foraging activity was positively correlated with walking speed within 4 hours (partial correlation coefficients from autoregressive models > 0.5). When temperatures were highest (observation 2), foraging rate was correlated with walking speed within 8 hours.

## Discussion

We found that *L*. *humile* colonies foraged from winter aggregations during all day and night hours. Other work has shown moderate levels of wintertime foraging by *L*. *humile* in northern California [23] and North Carolina, USA [41]. Our observations suggest that tree structure could facilitate continuous *L*. *humile* foraging, as in [21, 41], perhaps by offering dry microenvironments in bark cracks or on the underside of branches that protect forgers from rain. Foraging activity of the *L*. *humile* colonies increased from an early morning low until a peak in the afternoon, and then gradually declined until the following morning (Figs 2 and 3). A similar pattern of circadian oscillations in winter foraging by *L*. *humile* was found during a 24-hour period at four trees in a southern California citrus grove [22], and the alignment with our findings may suggest a relatively conserved daily activity cycle in winter.

Argentine ant recruitment, in response to trail pheromone, is primarily known from experiments investigating exploratory behavior [42, 43]. In our study, colonies were not exploring a new habitat, but instead apparently harvesting the honeydew of scale insects, a relatively persistent resource [7, 22, 40, 41]. All colonies maintained a ratio of about one to one of foragers traveling up to foragers traveling down a tree (Fig 3), and rates of outgoing foragers were also correlated with rates returning to the nest within the previous hour (Fig 4). Recent work indicates that the volume of *L*. *humile* foragers on bidirectional trails is altered in response to changing resource abundance and quality [44]. We hypothesize that, as in other species (e.g. the harvester ant *Pogonomyrmex barbatus*) [45], returning foragers may stimulate outgoing foragers to leave the nest.

*Linepithema humile* foraging trails dissolve when trail pheromone concentrations are reduced to levels that are undetectable by workers [46]. During intense rainstorms, *L*. *humile* left foraging trails and aggregated in sheltered locations on the trees–perhaps because chemical trails were obscured when rain water saturated the tree trunks. Similar behavior during rain has also been observed in the leaf-cutting ant *Atta cephalotes*, when workers abandon the foraging trail in search of dry locations on nearby trees [47, 48]. Behavior similar to shelter-seeking on foraging trails may be involved in the formation of winter aggregations. In choosing aggregation sites, colonies react to pervasive wet and cold conditions, on a larger spatial and temporal scale, by condensing many nests and a large trail network to one or two sheltered nest sites [3, 24].

The temporal partitioning of nutritional resources within ant communities is influenced by competition [15], and this can limit the distribution of *L*. *humile* [35]. *Prenolepis imparis* forages on both trees and the ground in large numbers during winter in northern California [39]. However, we observed this native species only at very low levels on the trees where *L*. *humile* colonies were aggregated. Although *P*. *imparis* possesses a chemical defense that can harm *L*. *humile* aggressors [39], and can reduce habitat colonization by *L*. *humile* [38], it appears that these small numbers of *P*. *imparis* ants did not deter *L*. *humile* foraging (Fig 5B).

Forager walking speed was positively associated with temperature and showed a negative trend with respect to humidity (Fig 5A), as is common in colonial poikilotherms [9–11]. During the low wintertime temperatures we observed, the average rate at which walking speed increased with temperature (0.10 cm s^-1^°C^-1^) was half that reported in *L*. *humile* under higher summer temperatures (0.20 cm s^-1^°C^-1^ at 25.2–33.8°C) [10]. Peaks in walking speed preceded peaks in foraging activity, with the time elapsed between maximum daily speed and foraging rate longer during warmer conditions than cooler conditions (Fig 2). While walking speed exhibited a mild positive parabolic relationship with temperature, foraging rate exhibited a strong negative parabolic relationship with temperature (Fig 5A and 5B). Thus, foraging activity and locomotion rate differed in their relation to temperature.

*Linepithema humile* is most likely to occur where mean daily temperatures in mid-winter range between 7 and 14°C [31]. We found that in winter, colonies appeared to prefer to forage most from 10–15°C, even though the maximum daily temperatures ranged from 17.7 to 24.05°C (Fig 2). This contrasts with the high rates of *L*. *humile* foraging at higher temperatures during the summer polydomous phase [22–25]. However, our results do not show whether the surge in foraging effort from 10–15°C reflects temperature preference, as these thermal conditions briefly occurred in morning hours with no spike in foraging effort. It may be that foraging rate is related to a circadian rhythm that happens to align with a narrow temperature range. Although it is likely that a variety of factors are at play, the 10–15°C thermal window may have provided an improved opportunity, metabolic [11–14, 29] or environmental [44, 49], for *L*. *humile* colonies to forage on hemipteran honeydew during a time of reduced resource availability [6, 7].

## Methods and materials

### Study area

The study was conducted in a 5.15-acre cultivated woodland bordered by footpaths, buildings, and a parking lot on Stanford University’s main campus, CA, USA (37°25'47"N by 122°10'14"W) (Fig 1A). This woodland is mostly composed of *Quercus agrifolia* (Coast live oak), with *Sequoia sempervirens* (Coast redwood), *Olea sp*. (Olive), and *Quercus douglasii* (Blue oak) in the overstory. Several *Aesculus californica* (California buckeye), *Rhamnus sp*. (Buckthorn), and *Ceanothus thyrsiflorus* (Blueblossom ceanothus) grow under the main canopy. The understory is dominated by non-native annual European grasses, and includes scattered pedestrian paths, leaf litter, and wood chips. There was moderate foot, bicycle, and service vehicle traffic throughout the study, but no direct impact of human activity on the trees the ants were using.

In January 2016, we identified three winter aggregations of *L*. *humile* colonies (Fig 1A). Colonies 2 and 3 were approximately 20 m apart and Colony 1 was about 100 m away from 2 and 3. Although trails can extend up to 43 m from a winter aggregation [1], in observations from January to April 2016, we never observed trails linking any of the three aggregations, so we considered the 3 aggregations to be separate colonies [2, 50]. Each colony's aggregation nest was at the base of a *Q*. *agrifolia* tree exposed to southern light. All three colonies had established a bidirectional foraging trail leading from the nest, up along the trunk, and into the tree canopy.

### Environmental conditions

To monitor environmental conditions during our behavioral observations, we arranged and affixed a DHT22 temperature and humidity sensor, CdS photoresistor, Arduino UNO processor, SD card, and 9V alkaline battery inside a transparent, cylindrical plastic tube using hot glue, hung 2 m off the ground underneath a southeast-facing branch in tree 2 (Fig 1A). We recorded ambient temperature (°C), relative humidity (0–100%), and light level every 5 seconds for the duration of all observation periods. We also measured the surface temperature of tree trunks where foraging trails were located during the second observation (05:00 on 2/22–07:00 on 2/23) to compare with air temperature recorded by the data logger (Ryobi ZRIR001 Non-Contact Infrared Thermometer, Solar Wide Industrial Ltd., Hong Kong). We recorded the number of *P*. *imparis*, the only other ant species we found in the study area, in a specific region of all tree trunks (trunk area, Fig 1B) at the beginning of all observations.

### Colony behavior

The foraging trails of the three *L*. *humile* colonies were simultaneously monitored in three sets of observations in winter 2016. Observation 1 was made from 05:00 PST on 2/1–07:00 on 2/2 (27 h); observation 2 from 05:00 on 2/22–07:00 on 2/23 (27 h); and observation 3 from 06:00–23:00 on 3/14 (17 h). To minimize disturbance, observations at night were made using the red light setting on Fred LED Headlamps (Princeton Tec^®^, USA). At each colony, a line was marked perpendicular to the foraging trail and tree trunk 1.0 m up the trunk from the main nest entrance to measure foraging rate. Just below this rate line, we marked a second 10.0 cm line, the speed line, parallel to the foraging trail (Fig 1B). We made behavioral measurements at the rate and speed lines each hour at each aggregation. All foraging trail locations, and thus rate and speed lines, remained consistent throughout the study. Foraging was estimated by counting the number of *L*. *humile* ants that passed the rate line travelling to and from nests for 16 sequential 30 s intervals during each observation. Ant walking speed was measured by recording the time it took 1–10 individual foragers headed both directions to travel the length of the speed line.

### Statistical analysis

All analyses were conducted in R [51] or MATLAB [52], were two-tailed, and met assumptions unless otherwise noted. Measurements of physical variables taken every 5 seconds were averaged for each aggregation’s hourly observation period. To evaluate how well ambient temperature predicted tree surface temperature, we used linear regressions for each tree with ambient and surface temperature on 2/22–2/23 as the independent and dependent variables, respectively. We found for each observation period the mean of the foraging rate (number of foragers s^-1^) and forager speed (cm s^-1^) counts in both directions.

To capture general trends of association despite differences among colonies, hourly foraging rates and speeds were normalized on a 0–1 scale within colonies and days. The highest foraging rate and speed for a given colony on a given day would be 1, and the lowest rate and speed 0. The abiotic conditions under which the highest foraging rates and forager speeds occurred were investigated by averaging the normalized (0–1 scale) foraging rate and forager speed of all colonies on the coldest and warmest observations (1 and 2, respectively). We then plotted these values (± 1SD) against linearly interpolated temperature and humidity data (Fig 2).

To illustrate the temporal pattern of foraging activity, we plotted the mean foraging rate in each direction over time (Fig 3). We examined the correlation between foraging rate up and down the trees by calculating shifts in the timing of association. The partial correlation coefficients between normalized (0–1 scale) hourly colony foraging rates in both directions were calculated for each colony using autoregressive models, with values above 0.5 or below -0.5 considered to be significant positive and negative correlation, respectively. Correlation of these two variables was examined at shifts of -12 to 12 hours. If outgoing foragers tended to leave the nest shortly after returning foragers entered the nest, partial correlation coefficients between foraging rates up and down would be significantly positive with no or little time shift (Fig 4).

To examine how environmental parameters were related to *L*. *humile* foraging rate and forager walking speed up trees, which were non-independent measures, we performed both linear and quadratic mixed effects analyses with foraging rate or walking speed as dependent variables and ambient temperature, relative humidity, light level, and *P*. *imparis* abundance as independent variables. Environmental predictors were the fixed effects, and to account for variation among colonies and trees, by-colony and by-day intercepts and slopes for the influence of environmental predictors on speed and foraging were random effects. Prior to running the models, both independent and dependent variables were log-transformed so that all data would be on a similar scale, thus enhancing the signal of non-dominant data. We performed one linear and one quadratic mixed effects analysis for each dependent variable as a function of all independent variables. For both pairs of linear and quadratic models, we used ANOVA to perform a likelihood ratio test (Chi-square) to determine if the quadratic model had explanatory value over the linear model (alpha = 0.05). If the reduction in residual sum of squares was statistically significant, we reported results from the quadratic model. In both cases, the quadratic models were used. After model selection, we identified outlying data points based on quantile-quantile plots of the residuals. If outliers were present (points well beyond 95% CIs), we ran models with and without these data. Finding similar direction and significance of effects to models with outliers included, we report only model results with these data removed (3 and 6 data points were removed from the foraging rate and forager speed model, respectively). P-values for each predictor were determined using likelihood ratio tests to compare the model with each effect against the model without it. Coefficients (± SE) and p-values for each predictor-response comparison from mixed effects analyses are identified based on predictor and significance (a Bonferroni correction reduces alpha to 0.013) (Fig 5). Two independent variables, temperature and humidity, exhibited a relatively high degree of correlation (0.83) and thus may have led to type II error. We re-ran both analyses with temperature or humidity omitted from the fixed effects to assess the potential for inaccuracy in the respective coefficients and p-values (Fig 5).

To determine if foraging rate was associated with walking speed, and if this association depended on temperature, we compared the correlation between foraging rate and walking speed up trees on the coldest and warmest observations (1 and 2, respectively). The partial correlation coefficients between normalized (0–1 scale) hourly foraging rate and walking speed were calculated for each colony using autoregressive models, as described above. If colonies foraged most when workers were travelling most rapidly, partial correlation coefficients would be significantly positive with little or no time shift.

## Supporting information

S1 Data The dataset recorded and analyzed during the current study. (CSV)

## References

[1] Flanagan TP, Pinter-Wollman NM, Moses ME, Gordon DM. 2013 Fast and flexible: Argentine ants recruit from nearby trails. *PLoS One*. 8, e70888 10.1371/journal.pone.0070888 23967129 PMC3743883

[2] Heller NE, Ingram KK, Gordon DM. 2008 Nest connectivity and colony structure in unicolonial Argentine ants. *Insectes Soc*. 55, 397–403.

[3] Markin GP. 1970 The seasonal life cycle of the Argentine ant, *Iridomyrmex humilis* (Hymenoptera: Formicidae), in southern California. *Ann*. *Entomol*. *Soc*. *Am*. 63, 1238–1242.

[4] Reuter M, Balloux F, Lehmann L, Keller L. 2001 Kin structure and queen execution in the Argentine ant *Linepithema Humile*. *J*. *Evol*. *Biol*. 14, 954–958.

[5] Abril S, Oliveras J, Gómez C. 2008 Effect of seasonal dynamics on queen densities of the Argentine ant (*Linepithema Humile*) (Hymenoptera: Formicidae) in an invaded natural area of the NE Iberian Peninsula. *Sociobiol*. 51, 645–654.

[6] Mooney HA. 1983 Carbon-gaining capacity and allocation patterns of mediterranean-climate plants in Mediterranean-Type Ecosystems: Origin and Structure (eds. Castri F. & Mooney H. A.) Springer Berlin Heidelberg, pp 103–119.

[7] Rowles AD, Silverman J. 2009 Carbohydrate supply limits invasion of natural communities by Argentine ants. *Oecologia*. 161, 161–171. 10.1007/s00442-009-1368-z 19452171

[8] Gordon DM. 2010 Ant encounters: interaction networks and colony behavior Princeton University Press.

[9] Shapley H. 1920 Thermokinetics of *Liometopum apiculatum* Mayr. *Proc*. *Nat*. *Acad*. *Sci*. *U*. *S*. *A*. 6, 204–211. 16576491 10.1073/pnas.6.4.204 PMC1084464

[10] Shapley H. 1924 Note on the thermokinetics of Dolichoderine ants. *Proc*. *Nat*. *Acad*. *Sci*. *U*. *S*. *A*. 10, 436–439. 16576855 10.1073/pnas.10.10.436 PMC1085745

[11] Hurlbert AH, Ballantyne F, Powell S. 2008 Shaking a leg and hot to trot: the effects of body size and temperature on running speed in ants. *Ecol*. *Entomol*. 33, 144–154.

[12] Traniello JF, Fujita MS, Bowen RV. 1984 Ant foraging behavior: ambient temperature influences prey selection. *Behav*. *Ecol*. *Sociobiol*. 15, 65–68.

[13] Huey RB, Kingsolver JG. 1989 Evolution of thermal sensitivity of ectotherm performance. *Trend*. *Ecol*. *Evolut*. 4, 131–135. 10.1016/0169-5347(89)90211-5 21227334

[14] Traniello JF. 1989 Foraging strategies of ants. *Ann*. *Rev*. *Entomol*. 34, 191–210.

[15] Crist TO, MacMahon JA. 1991 Foraging patterns of *Pogonomyrmex occidentalis* (Hymenoptera: Formicidae) in a shrub–steppe ecosystem: the roles of temperature, trunk trails, and seed resources. *Environ*. *Entomol*. 20, 265–275.

[16] Cerdá X, Retana J, Cros S. 1998 Critical thermal limits in Mediterranean ant species: trade-off between mortality risk and foraging performance. *Funct*. *Ecol*. 12, 45–55.

[17] Ruano F, Tinaut A, Soler JJ. 2000 High surface temperatures select for individual foraging in ants. *Behav*. *Ecol*. 11, 396–404.

[18] Jayatilaka P, Narendra A, Reid SF, Cooper P, Zeil J. 2011 Different effects of temperature on foraging activity schedules in sympatric Myrmecia ants. *J*. *Exp*. *Biol*. 214, 2730–2738. 10.1242/jeb.053710 21795570

[19] Nonacs P, Dill LM. 1990 Mortality risk vs. food quality trade‐offs in a common currency: ant patch preferences. *Ecology* 71, 1886–1892.

[20] Abrams PA. 1991 Life history and the relationship between food availability and foraging effort. *Ecology* 72, 1242–1252.

[21] López F, Serrano JM, Acosta FJ. 1992 Temperature-vegetation structure interaction: the effect on the activity of the ant Messor barbarus in Quercus ilex L. Ecosystems: Function, Dynamics and Management (eds. Romaine F. & Terradas J.) Springer Netherlands pp. 119–128.

[22] Markin GP. 1970 Foraging behavior of the Argentine ant in a California citrus grove. *J*. *Econ*. *Entomol*. 63, 740–744.

[23] Human KG, Weiss S, Weiss A, Sandler B, Gordon DM. 1998 Effects of abiotic factors in the distribution and activity of the invasive Argentine ant (Hymenoptera: Formicidae). *Environ*. *Entomol*. 27, 822–833.

[24] Heller NE, Gordon DM. 2006 Seasonal spatial dynamics and causes of nest movement in colonies of the invasive Argentine ant (*Linepithema humile*). *Ecol*. *Entomol*. 31, 499–510.

[25] Carpintero S, Retana J, Cerdá X, Reyes-López J, Arias de Reyna L. 2007 Exploitative strategies of the invasive Argentine ant (*Linepithema humile*) and native ant species in a southern Spain pine forest. *Environ*. *Entomol*. 36, 1100–1111. 18284734 10.1603/0046-225x(2007)36[1100:esotia]2.0.co;2

[26] Thomas ML, Holway DA. 2005 Condition‐specific competition between invasive Argentine ants and Australian Iridomyrmex. *J*. *Animal*. *Ecol*. 74, 532–542.

[27] Holway DA. 1999 Competitive mechanisms underlying the displacement of native ants by the invasive Argentine ant. *Ecology* 80, 238–251.

[28] Menke SB, Fisher RN, Jetz W, Holway DA. 2007 Biotic and abiotic controls of Argentine ant invasion success at local and landscape scales. *Ecology* 88, 3164–3173. 18229850 10.1890/07-0122.1

[29] Jumbam KR, Jackson S, Terblanche JS, McGeoch MA, Chown SL. 2008 Acclimation effects on critical and lethal thermal limits of workers of the Argentine ant, *Linepithema humile*. *J*. *Insect Physiol*. 54, 1008–1014. 10.1016/j.jinsphys.2008.03.011 18534612

[30] McGrannachan CM, Lester PJ. 2013 Temperature and starvation effects on food exploitation by Argentine ants and native ants in New Zealand. *J*. *Applied Entomol*. 137, 550–559.

[31] Hartley S, Harris R, Lester PJ. 2006 Quantifying uncertainty in the potential distribution of an invasive species: climate and the Argentine ant. *Ecol*. *Lett*. 9, 1068–1079. 10.1111/j.1461-0248.2006.00954.x 16925656

[32] Menke SB, Holway DA, Fisher RN, Jetz W. 2009 Characterizing and predicting species distributions across environments and scales: Argentine ant occurrences in the eye of the beholder. *Global Ecol Biogeog*, 18, 50–63.

[33] Roura-Pascual N, Hui C, Ikeda T, Leday G, Richardson DM, Carpintero S, et al 2011 Relative roles of climatic suitability and anthropogenic influence in determining the pattern of spread in a global invader. *Proc Nat Acad Sci U*. *S*. *A*., 108, 220–225. 10.1073/pnas.1011723108 PMC3017164 21173219

[34] Gordon DM, Heller NE. 2014 The invasive Argentine ant *Linepithema humile* (Hymenoptera: Formicidae) in northern California reserves: from foraging behavior to local spread. *Myrmecol*. *News*. 19, 103–110.

[35] Vonshak M, Gordon DM. 2015 Intermediate disturbance promotes invasive ant abundance. *Biol*. *Conserv*. 186, 359–367.

[36] Tschinkel WR. 1987 Seasonal life history and nest architecture of a winter-active ant, *Prenolepis imparis*. *Insectes Soc*. 34, 143–164.

[37] Human KG, Gordon DM. 1996 Exploitation and interference competition between the invasive Argentine ant, *Linepithema humile*, and native ant species. *Oecologia* 105, 405–412. 10.1007/BF00328744 28307114

[38] Fitzgerald K, Gordon DM. 2012 Effects of vegetation cover, presence of a native ant species, and human disturbance on colonization by Argentine ants. *Conserv*. *Biol*. 26, 525–538. 10.1111/j.1523-1739.2012.01836.x 22533673

[39] Sorrells TR, Kuritzky LY, Kauhanen PG, Fitzgerald K, Sturgis SJ, Chen J, et al 2011 Chemical defense by the native winter ant (*Prenolepis imparis*) against the invasive Argentine ant (*Linepithema humile*). *PLoS One*. 6, e18717 10.1371/journal.pone.0018717 21526231 PMC3079705

[40] Bartlett BR. 1961 The influence of ants upon parasites, predators, and scale insects. *Ann*. *Entomol*. *Soc*. *Am*. 54, 543–551.

[41] Brightwell RJ, Silverman J. 2011 The Argentine ant persists through unfavorable winters via a mutualism facilitated by a native tree. *Environ*. *Entomol*. 40, 1019–1026. 10.1603/EN11038 22251714

[42] Deneubourg JL, Aron S, Goss S, Pasteels JM. 1990 The self-organizing exploratory pattern of the argentine ant. *J*. *Insect Behav*. 3, 159–168.

[43] Reid CR, Sumpter DJ, Beekman M. 2011 Optimisation in a natural system: Argentine ants solve the Towers of Hanoi. *J*. *Exp*. *Biol*. 214, 50–58. 10.1242/jeb.048173 21147968

[44] Latty T, Holmes MJ, Makinson JC, Beekman M. 2017 Argentine ants (*Linepithema humile*) use adaptable transportation networks to track changes in resource quality. *J*. *Exp*. *Biol*. 220, 686–694. 10.1242/jeb.144238 28202653

[45] Gordon DM, Holmes S, Nacu S. 2008 The short-term regulation of foraging in harvester ants. *Behav*. *Ecol*. 19, 217–222. 10.1093/beheco/arq218 PMC3071749 22479133

[46] Suckling DM, Peck RW, Stringer LD, Snook K, Banko PC. 2010 Trail pheromone disruption of Argentine ant trail formation and foraging. *J*. *Chem*. *Ecol*. 36, 122–128. 10.1007/s10886-009-9734-1 20077128

[47] Hodgson ES. 1955 An ecological study of the behaviour of the leaf-cutting ant *Atta cephalotes*. *Ecology*. 36, 293–304.

[48] Farji-Brener AG, Dalton MC, Balza U, Courtis A, Lemus-Domínguez I, Fernández-Hilario R, et al 2018 Working in the rain? Why leaf-cutting ants stop foraging when it’s raining. *Insectes Soc*. 1–7.

[49] Grover CD, Dayton KC, Menke SB, Holway DA. 2008 Effects of aphids on foliar foraging by Argentine ants and the resulting effects on other arthropods. *Ecol*. *Entomol*. 33, 101–106.

[50] Ellis S, Procter DS, Buckham-Bonnett P, Robinson EJH. 2017 Inferring polydomy: a review of functional, spatial and genetic methods for identifying colony boundaries. *Insectes Soc*. 64, 19–37. 10.1007/s00040-016-0534-7 28255180 PMC5310590

[51] R Core Team. 2015 R: A language and environment for statistical computing. https://www.R-project.org/.

[52] The MathWorks, Inc. 2012 MATLAB and Statistics Toolbox Release.

